# Moxifloxacin-Induced Peripheral Neuropathy: A Rare Side Effect of Fluoroquinolone Therapy in Tuberculosis Management

**DOI:** 10.7759/cureus.103314

**Published:** 2026-02-09

**Authors:** Dillon Prus, Robert Lenox

**Affiliations:** 1 Department of Medicine, State University of New York (SUNY) Upstate Medical University, Syracuse, USA; 2 Tuberculosis Control Clinic, Onondaga County Health Department, Syracuse, USA

**Keywords:** adverse effect, fluoroquinolones, moxifloxacin, peripheral neuropathy, tuberculosis

## Abstract

Moxifloxacin (MXF) is a cornerstone of the newly recommended four-month 2HPMZ/2HPM regimen for drug-susceptible pulmonary tuberculosis (TB). However, potential side effects such as cumulative neurotoxicity remain poorly characterized in clinical practice. Here, we report a rare case of peripheral neuropathy (PN) occurring after 17 weeks (four months) of MXF treatment in a 60-year-old Vietnamese man treated for drug-sensitive pulmonary TB. Following early isoniazid-induced hepatotoxicity, the patient's regimen was modified to include MXF 400 mg daily, notably without other neurotoxic agents such as linezolid or ethambutol. After 17 weeks of MXF therapy, the patient developed symmetrical stocking-glove PN. Extensive workup, including hemoglobin A1c and folate levels, ruled out common metabolic and nutritional etiologies. Cessation of MXF and pyridoxine supplementation led to gradual symptom improvement with full resolution after four months. While FDA warnings emphasize the often-rapid onset of fluoroquinolone-associated nerve damage within days of initiation, this case illustrates a delayed, cumulative toxicity profile that aligns with recent epidemiological evidence. As global TB guidelines shift toward four-month MXF regimens, this report highlights a critical safety consideration for clinicians and the importance of routine neurological screening throughout the entire treatment course to detect early signs of toxicity and prevent the development of potentially permanent PN.

## Introduction

Moxifloxacin (MXF) is a potent fluoroquinolone (FQ) that exhibits broad-spectrum antibiotic activity, especially for respiratory pathogens such as *Mycobacterium tuberculosis* [1]. It has recently become a first-line treatment for tuberculosis (TB) following Study 31/A5349, which established the 17-week (four-month) daily regimen (2HPMZ/2HPM) as noninferior to the standard six-month course for drug-susceptible pulmonary TB [2,3]. While generally well-tolerated, common side effects of MXF include diarrhea, nausea, vomiting, headache, dizziness, and rash [1,4]. MXF also carries a class-wide risk of disabling and potentially permanent peripheral neuropathy (PN), evidenced by the FDA’s warning in 2013 [5].

Neuropathy in the context of TB is traditionally associated with isoniazid (INH), due to pyridoxine depletion, or ethambutol (EMB). Other standard agents, such as rifapentine and pyrazinamide (PZA), have not been significantly linked to PN [6]. However, other nonstandard agents used in multidrug-resistant TB, such as linezolid, have been associated with PN [7]. While the FDA warning often emphasizes a rapid onset of FQ-induced nerve damage within the first week of therapy [5], newer epidemiological data suggest a cumulative toxicity profile. Morales et al. estimated that the risk of PN increases by 3% for each additional day of FQ exposure [8]. Furthermore, recent pharmacovigilance analyses of the FDA Adverse Event Reporting System database found that MXF-containing regimens demonstrated a significantly higher statistical signal for PN than levofloxacin-based protocols [9]. Here, we report a rare case of sensory PN occurring at the completion of 17 weeks of MXF therapy in a 60-year-old man, in the absence of other neurotoxic agents.

## Case presentation

A 60-year-old Vietnamese man presented with drug-sensitive pulmonary TB. His chest X-ray showed extensive cavitation. The patient was started on the standard RIPE protocol: PZA 1,000 mg/day (20 mg/kg), rifampin (RIF) 600 mg/day, INH 300 mg, and EMB 800 mg/day (15 mg/kg) with pyridoxine 50 mg daily. He was not started on a four-month regimen because rifapentine was unavailable at the time.

After 15 days of treatment, the patient reported abdominal pain, decreased appetite, arthralgia, chest pain, headache, dizziness, joint pain, nausea, malaise, shortness of breath, and weight loss. Upon exam, he had scleral icterus, and lab values showed an elevated alanine aminotransferase at 187 U/L (reference: 9-46 U/L) (Table 1). INH, RIF, and PZA were discontinued. RIF and PZA were added back sequentially as the patient's abdominal pain and scleral icterus improved. Since the resumption of RIF and PZA did not cause further hepatotoxicity, INH was identified as the causative agent. Consequently, both INH and pyridoxine were discontinued. MXF 400 mg daily was substituted for the INH, and EMB was also discontinued to simplify the regimen.

**Table 1:** Laboratory studies ALT: alanine aminotransferase; INH: isoniazid

Laboratory test	Result	Reference range
Hgb A1c	4.8%	<5.7%
Folate	13.7 ng/mL	Normal >5.4 ng/mL, borderline 3.4-5.4 ng/mL, low <3.4 ng/mL
Pretreatment ALT	33 U/L	9-46 U/L
ALT after INH	187 U/L	9-46 U/L

After 17 weeks of MXF treatment, the patient reported paresthesia in his fingers and hands. Upon examination, there was a stocking-glove distribution of hypoesthesia bilaterally to pinprick. The rest of his neurologic examination, including motor strength, was normal. MXF was discontinued, and the patient was started on pyridoxine 50 mg daily and restarted on EMB to complete his TB therapy. Notably, hemoglobin A1C and folate levels were within normal limits (Table 1). His symptoms began to improve after one month, and the PN resolved fully after four months.

## Discussion

PN is commonly associated with TB and its treatment. In addition to INH and EMB, several comorbidities often seen in TB can also lead to PN, such as HIV, diabetes mellitus, hypothyroidism, malnutrition, and alcohol use [6]. None of these were present in this case. As the PN developed only after starting MXF for 17 weeks, it is highly probable that the medication was the cause. The timeline of the patient's symptoms is also important to note, as 17 weeks corresponds to the length of the four-month regimen for drug-resistant TB [2,3].

To objectively assess causality, we applied the Naranjo Adverse Drug Reaction Probability Scale [10]. The patient's score was 6 (calculated based on previous reports of FQ-induced PN, symptoms appearing after drug administration, improvement upon withdrawal, and the absence of alternative causes), indicating a "probable" relationship between MXF and the patient's symptoms.

Previous studies have demonstrated that oral FQ therapy significantly increases the relative incidence of PN. The absolute risk with current oral FQ exposure was 2.4 per 10,000 patients per year of use [8]. While many clinicians associate PN with INH, the 17-week gap between the INH discontinuation and symptom onset in this patient makes a delayed INH reaction unlikely, especially given the prompt resolution of his symptoms following MXF cessation.

This patient's post-MXF PN differs from previous reports in several notable ways. In the FDA's 2013 updated warning about PN with the use of FQs, the organization noted the rapid onset of neurologic symptoms, sometimes presenting "within days" after starting the drug [5]. Here, the patient's symptoms did not present until after 17 weeks of treatment, highlighting the potential cumulative effects of the drug. This supports a more recent study, which found that the PN increased by approximately 3% for each additional day of current FQ exposure [8]. Additionally, while recent reports note the development of PN in patients with extensively drug-resistant and multidrug-resistant TB treated with drug regimens including MXF, they point to linezolid as a potential culprit [11,12]. Here, linezolid was absent in the treatment protocol, suggesting that MXF may be a contributing factor in PN in TB patients.

The exact mechanism of MXF-induced PN is not fully understood. While one study suggested axonal degeneration with secondary breakdown of the myelin sheath [13], more recent evidence points toward mitochondrial dysfunction. FQs have been shown to inhibit mitochondrial topoisomerase II, leading to oxidative stress and depletion of mitochondrial DNA in a range of tissues. Interestingly, the specific topoisomerase II inhibited by FQs, Top2β, is present in neural mitochondria at far higher levels than in other tissues, which may explain the greater susceptibility of peripheral nerves to mitochondrial depletion and the patient's purely sensory stocking-glove presentation [14]. Folate deficiency was also considered to be a possibly enabling factor for FQ-induced neuropathy [13]. However, in our case, the patient’s folate level was normal (Table 1).

Treatment of MXF-induced PN begins with the discontinuation of the offending medication. While we prescribed pyridoxine 50 mg/day, which has previously been recommended for FQ-induced PN [6], its benefit is unclear here, as the patient had no clear etiology for B6 deficiency at the time of PN onset. It is, therefore, most likely that the PN was reversible primarily due to the cessation of MXF.

## Conclusions

This report documents a rare case of late-onset PN, specifically associated with a 17-week course of MXF for drug-susceptible TB, that resolved upon cessation of the offending agent. To our knowledge, this is the first clinical report identifying MXF as the primary neurotoxic agent after 17 weeks of treatment, the same length as the 2HPMZ/2HPM regimen, in the absence of other common neurotoxic drugs such as linezolid. While historical regulatory warnings focus on a rapid onset within days of initiation, our case illustrates a cumulative toxicity profile consistent with epidemiological evidence that the risk of PN increases with each additional day of FQ exposure. As MXF becomes a cornerstone of shortened therapy for drug-sensitive TB, clinicians must recognize the risk of and look out for PN throughout the treatment course. Incorporating routine neurological assessments into every clinical visit may help detect early toxicity and prevent the development of potentially permanent PN.
